# GPs’ experience of difficult decisions in people with dementia who have an acute illness: a qualitative, semi-structured interview study

**DOI:** 10.3399/BJGPO.2024.0074

**Published:** 2025-02-26

**Authors:** Samuel Lassa, Chris Burton, Jon M Dickson

**Affiliations:** 1 Royal Primary Care, Ashgate Research Unit, Chesterfield Royal Hospital NHS, Chesterfield, UK; 2 School of Medicine & Population Health, Sheffield Centre for Health and Related Research, University of Sheffield, Sheffield, UK

**Keywords:** General practitioners, Decisions, Dementia, Acute disease, Primary healthcare

## Abstract

**Background:**

GPs are often required to make decisions about the management of acute illness in people living with dementia. These decisions are often complex and involve multiple informants.

**Aim:**

We aimed to explore how GPs made decisions about acute illness in people with dementia using a micropolitics approach.

**Design & setting:**

Qualitative, semi-structured interviews with 13 GPs with a range of years of experience working in South Yorkshire, UK.

**Method:**

Interviews were conducted by phone. Interviews focused on GPs’ accounts to reflect their own perceptions and choices as portrayed to the interviewer. The analysis used the lens of micropolitics in the analysis and interpretation of the themes, with a focus on decisions between GP, patient, family and carers, and other health and social care providers about the management of acute illness in a person with dementia.

**Results:**

The results showed that GPs act as street-level bureaucrats while carrying out their role, using discretion during decision-making in an environment characterised by uncertainties and work pressures. In addition, GPs use the ‘soft power’ skills of diplomacy, such as negotiation, persuasion, and engagement, in navigating difficult decision-making situations, while building relationships and partnerships with various actors in the health system.

**Conclusion:**

GPs possess and express power, and influence decision-making in people living with dementia when navigating biomedical, social, and psychological factors. This power comes in the form of soft power (street-level diplomacy) and the more formal power of street-level bureaucracy.

## How this fits in

Negative portrayals by the media depict GPs’ professional power as a barrier to health policy reform. Evidence from this study shows that, rather than subverting policies and government directives, GPs smooth the way for policy implementation through diplomacy. This finding contrasts with claims made in the new public management literature that professionals have had too much control of public services and are more focused on enhancing their professional status and rewards than actually providing quality and effective service to citizens.

## Introduction

Dementia is an increasing problem and in the UK an estimated 944 000 people are currently living with dementia.^1^ Although dementia is not in itself an acute medical condition, it is associated with a large number of emergency hospital admission in older persons. An estimated 42% of unplanned acute hospital admissions among those aged 70 years and above involve patients with dementia, amounting to 25% of hospital bed-days.^2^ The majority of these admissions relate to acute illnesses such as respiratory and urinary tract infections.^3^ However, acute hospital care carries additional risks for people with dementia and acute illness: hospital admissions increase their risk of agitation, delirium, depression, cognitive decline, and life-threatening hospital-acquired infections.^4^

Decisions about hospital admission for acute illness in people with dementia are often difficult. While some decisions are covered by advance care plans (ACP), which are used to communicate the patient’s wishes in advance for when they lose capacity, in some acute illness situations the various treatment options available may not have been discussed with patients and the ACP may not be directly applicable to the current problem. This leads to a decision-making dilemma which is often resolved by either the next of kin or the health professional, typically a GP.^5^ These decisions are difficult as they involve multiple parties, limited or absent capacity to make a decision by the patient, and incomplete guidance from the ACP. They become more difficult decisions when the health professional has no prior personal knowledge of the patient or the case. As GP assessment and admission is one of the pathways to acute hospital care, it is important to understand how GPs make difficult decisions about acute care for people with dementia who have intercurrent acute illness. Research into these difficult decisions is of prime importance in dementia care because of its impact on the equity of access to good quality medical care.^6^

Difficult decisions in the healthcare system can be analysed from the perspective of micropolitics.^7^ Micropolitics focuses on *‘those activities taken within organisations (health systems) to acquire, develop, and use power and other resources to obtain one’s preferred outcomes in a situation in which there is uncertainty or dissent’.* ^8^ This offers a useful perspective on difficult decisions by GPs around acute illness in people with dementia, where interactions between health practitioners, patients, and carers on the best way forward often require negotiation and compromise. This is particularly challenging when the patient is unable to communicate their needs as a result of dementia. Some authors have used the micropolitics lens, and related constructs of street-level bureaucrats or street-level diplomats, to examine some of these difficult decision making processes.^9–13^ According to Lipsky,^14^ street-level bureaucrats encounter a lot of work pressure and uncertainty because they are involved at the micro-level or grassroot level of policy implementation (see Table 1 for additional information). In the health system, the role GPs occupy is similar to Lipsky’s concept of street-level bureaucrats. However, with the changing health system landscape in the NHS, some commentators have argued the importance of more nuanced research exploring Lipsky’s and other concepts that describe the interactions of GPs and their patients.^15^

**Table 1.** Micropolitical analysis of health decisions

According to Lipsky, street-level bureaucrats encounter a lot of work pressure and uncertainty because they are involved at the micro- or grassroot level of policy implementation.^14^ At this micro-level, many uncertainties exist and the autonomy and discretion of street-level bureaucrats allow them to implement policies case by case. This discretionary power enables street-level bureaucrats to exercise substantial influence in the micropolitical network of decision making and autonomy to make appropriate decisions on behalf of the system they represent. According to Lipsky this is how street-level bureaucrats are able to link policy with the population in situations of complex decision making.^14^ However, with the changing health system landscape in the NHS, some commentators have argued that GPs’ decisions are substantially constrained by rules and policies.^26^ and unable to fully exercise these discretionary powers. Hence, more nuanced research is needed to explore other concepts that describe the interactions of GPs and their patients.

We aimed to understand how GPs made decisions about acute illness in people with dementia using the approach of micropolitics with a particular focus on situations where ACPs were not present or did not account for the situation at hand.

## Method

### Study design

We carried out a qualitative study of GPs’ accounts of making difficult decisions for acute illness in people with dementia. We invited GPs who had completed specialist training and regarded themselves as having made difficult decisions about acute illnesses for people with dementia within the last 2 years. We recruited GPs working in both in-hours and out-of-hours general practice in the South Yorkshire region. Invitations were through clinical and university networks, and used a mixture of purposive and snowballing sampling technique. The initial sampling was purposive to recruit a sample of GPs with diversity in length and type of experience. The invitation letters contained background information about the research team and the study goals. After the initial stage of purposive sampling, the snowballing technique was used to reach out to more GPs within the participants’ networks. In the process, 22 participants were approached via email. We aimed to obtain between 10 and 20 interviews, with a plan to stop data collection when no new substantial data were identified in two consecutive interviews.

### Interviews

We collected data using semi-structured interviews. These were conducted by phone by SL (a male GP academic clinical fellow, near the end of his training with an interest in primary care research) in a university office and lasted no more than 30 minutes. The content of the interview followed a topic guide, which had open-ended questions and was used flexibly. In order to focus the interviews on actual decisions,^16^ participants were asked in advance to reflect on one or more recent instances when they had to make a difficult decision for a patient with dementia and acute illness, particularly when it involved choices around admission to hospital. The interview guide was pilot tested with a non-participant independent GP who was not part of the research team. At the start of the interview the researcher asked the participant to describe one or more of the instances and then to elaborate on their decisions and the circumstances of the case. As interviews took place between July 2020 and April 2021, we also asked about how those difficult decisions were affected by the COVID-19 crisis. The informal flow of the interviews allowed participants to use other experiences to compare with the primary experience they were reflecting on. This enriched the quality of the data, as participants reflected on both favourable and unfavourable experiences during the interviews. Interviews were audio-recorded and subsequently transcribed for analysis. Rapport was established before audio recording and at the end of all interviews, participants were given an opportunity to reflect, make comments, offer suggestions, and give feedback to SL. There were no non-participants involved in the interviews. SL’s field notes were reviewed by SL, CB, and JMD regularly for contextualisation of the interview data.

### Participants and decisions

We interviewed 13 GPs (seven male and six female). Their experience ranged from 1–32 years post-CCT (Certificate of Completion of Training), with a median of 16 years. GPs held various roles, with seven partners, five associates or salaried doctors, and one locum. A total of 11 GPs worked only in-hours while two worked both in-hours and out-of-hours services. During the interviews, the GPs reflected on 21 instances of difficult decisions (16 involving care home residents and five involving patients living in their own home). Difficult decisions included admission to acute medical care (or not) for people with dementia and acute illnesses, and deterioration including infections (urinary tract and respiratory), falls, poor oral intake, complications of diabetes, progressive deterioration, and inability to manage at home. All decisions discussed involved patients with fluctuating mental capacity or no capacity to make decisions. Decisions such as when to admit or not admit were often complicated because the patients were new to the practice area, or had progressively deteriorated during the pandemic, as well as other non-medical issues such as personal conflicts between various parties.

### Analysis

Braun and Clarke’s six phases of thematic analysis were used in order to understand and interpret the interviews using a reflexive approach.^17^ Coding was conducted iteratively by SL and discussed in regular analysis meetings among the research team (CB and JMD). Coding was carried out in NVivo (version 12), through an iterative process refining the initial data into themes. Analysis took place in tandem with data collection so that later interviews could reflect emerging ideas. The themes were viewed through the street-level bureaucrat lens to elevate the themes from superficial ideas to three high level constructs, namely discretion, pressure of time, and uncertainty about translating policy and guidelines.^14^ Themes that were inconsistent or contradictory to the theoretical model of street-level bureaucrats were analysed independently, which produced the fourth theme of diplomacy.

## Results

### Overview of themes

We identified four high level construct key themes: discretion, pressure of time, uncertainty about translating policy and guidelines, and diplomacy. These are described in more detail below.

### Discretion

GPs used a substantial amount of discretion in the execution of their work. Whether participants were faced with best interest decisions in the absence of a next of kin during emergencies, withdrawing a patient’s existing medication, initiating palliative care, or determining a patient’s capacity to drive, GPs expressed their discretionary influence when using available information in the interpretation of existing guidelines. GPs described this skill as essential to carrying out their role in difficult situations, as guidelines and ACPs were insufficient if used solely in determining the final decisions to be made.

GPs expressed this power of discretion when they had to either draw on their ‘clinical experience and judgement rather than a protocol’, make judgements on whether to go against ACPs, or know when withdrawing medications from patients was in their best interests.

In some situations, GPs had to rely on their experience to make clinical judgements rather than protocols. In the scenario below the GP had to start a patient on end-of-life medications out of hours without any input from next of kin because they were unavailable:

*‘it was sort of much more difficult than any decision I had made before because I had to make the decision without someone who had looked after the patient.... I think these decisions are just made from yourself and from clinical experience and judgement rather than a protocol or tick box.’ (Interview 6, Initiating palliative care)*

In some situations, GPs have had to re-evaluate ACPs and consider if it would be for or against the best interest of the patient:

*‘But you can sometimes go against it* [ACP] *you know, if the situation changes or there is a condition, and you can go “no this is not what the care plan is about here”. So if they are in agony and in pain and they are riling about and really in distress or sometimes the fast track teams do not have enough capacity to take on new people and look after them. Then I think “hang on I can’t leave this patient in their home because we don’t have the right thing in place*”’ *(Interview 7, Interpreting an ACP)*

In the scenario below, the GP had to make the difficult decision in the patient’s best interest to stop all medications even though their condition was not 'end of life'. In this GP’s reflections, being assertive when faced with difficult decisions was a key characteristic of the GP role:

*‘You should not be scared. You have to tell the family honestly and openly about withdrawing the medications and explain to them why. You have to be able to tell them “These medications which I thought before were important are actually not needed any more”’ (Interview 1, Withdrawing medication)*

In summary, GPs have considerable discretion in clinical decision-making. From the data, GPs did find this skill essential in carrying out their role in difficult situations, as guidelines and ACPs were insufficient for sole use in determining the final decisions to be made.

### Working under pressure

Working under pressure as a theme describes the time pressures and limited resources that GPs encounter as they navigate through their work environment.

Our findings revealed that GPs’ translation of policies and guidelines is fraught with unexpected challenges and scenarios. Work pressures included limited time, insufficient resources, and a demanding workload when managing patients with dementia in acute situations, leading to feelings of frustration:

*‘So from a personal side there was that pressure of time, pressure of other work inevitably and the slight frustration of something called through which you could have dealt with when other staff were available.’ (Interview 4, Unwell care home resident)*

The theme ‘Working under pressure’ from this study typifies a street-level bureaucrat characteristic, where almost all the GPs felt the frustration of having to work with limited resources, while at the same time accepting that it was part of their role as a GP.

### Uncertainty about translating policies and guidelines

From the data, GPs found themselves in difficult situations when managing care homes under COVID uncertainties and, in some cases, dealing with the translation of the Mental Health Act when different health and social care professionals have conflicting mental capacity assessments.

Some of these uncertainties were more prominent during the pandemic when issues around hospital bed capacities, lack of personal protective equipment, and delays in service delivery were considered during decision-making. In one scenario, during a COVID outbreak in a care home, the responsible GP was faced with many uncertainties, which were made more complex with the lack of test kits:

*‘and there were a lot of things we had to contemplate. Like you know “Where to deliver best care?”, “whether hospital admissions?”, “Were we dealing with COVID or not?”...“What will happen if patients deteriorate?, would they be* '*end of life'?” It was all very unclear and uncertain.’ (Interview 3, Outbreak of infection in care home).*

In some instances, uncertainty about the translation of mental capacity assessments during best interest meetings involved conflicting ideas with other actors in the health and care system:

*‘And I had to chair some meetings and there was a meeting where there was a standoff about whether or not he had capacity on his health and wellbeing. And the health professionals felt he didn’t have capacity ... But social services felt he did have capacity when they saw him (Interview 5, Conflicting mental capacity assessments)*

Some GPs complained about the difficulty in getting input from the memory clinic during the pandemic, as an example, which was a major factor in delayed diagnosis of early dementia in primary care:

*‘I think the biggest challenge like with any other case is the delay in suspecting someone has dementia. Unfortunately, there is always delay because there are no resources for them to be seen very quickly and obviously because of the pandemic the delay has sort of gotten longer’ (Interview 12, Diagnosis of dementia)*

The quote above highlights how limited resources can be a source of uncertainty and challenge for GPs when executing their role. GP working environment and role is characterised by various rules, policies, legal procedures, and bureaucratic hurdles that they must in some way work with and towards; this is vital to the success of health programmes during situations of uncertainty.^11^

### Diplomacy

Even though our participants had some level of autonomy and discretion in some aspects of their interactions, in difficult situations they relied on their ability to persuade various people in the decision-making process to reach shared mutual goals. Most participants said they invested time and resources in building relationships because they found it useful for when a difficult decision had to be made in the future. Building trust in this way allowed GPs to use their soft power in influencing decisions:

*‘So my feeling of acute illness in dementia, is that when you feel someone is getting dementia you really need to get those relationships going early on and get that understanding because that makes the acute illness that much easier to manage.’ (Interview 5, Diagnosis of dementia in a patient).*

*'So I built up that kind of trust between myself as the health professional, the patient, and the daughter being the point of contact. So yes it certainly helps to know the patient feels you have their best interest at heart and they trust your decision making.’ (Interview 12, Recurrent illness in a dementia patient).*

In this study, GPs have used their position acting as facilitators during negotiations between various actors, creating an environment for collaborative decision-making. For example, there are instances where two relatives with a power of attorney holding differing views needed the GP to bring them to agree on a health decision:

*‘Almost like a negotiation in a sense. Almost like preparing a case for a difficult negotiation. And certain people in those negotiations felt strongly one way or the other… In a best interest meeting, you had to be careful. You know, checking where the personal values stopped and where the objective best interests of what the person would want starts.’ (Interview 5, Best interest meeting about a dementia patient)*

While in other instances, GPs were more effective when they sought collaboration and partnerships within the health system from other health professionals and the public:

*‘I know this family so well, and I was going in my head “I need to help them” ... But thank goodness I came across a really helpful fast response lady and we kept in contact through mobile which was the quickest way and it really helped ... she did a home visit and called me on my mobile at 8pm that evening.’ (Interview 10, Patient refusing admission).*

### Street-level bureaucracy or street-level diplomacy

Our analysis found that GPs act as street-level bureaucrats using their clinical authority during decision-making processes that involve biomedical, social, and psychological factors. It was apparent when GPs were faced with time pressures and situational uncertainty, especially during the COVID pandemic, they expressed discretion and autonomy in navigating those circumstances. This power of discretion enables street-level bureaucrats to *‘apply their own judgements when dealing with the needs and wishes of citizens’*.^18^ These themes are similar to those discussed in Lipsky’s concept, but only two street-level bureaucrat characteristics (discretion and autonomy) were identified in this study. Other themes revealed some characteristics not mentioned in Lipsky’s concept such as the use of ‘soft’ power to persuade and engage through ‘negotiations’ and ‘building of trust’. From the data, GPs during their interactions with actors used these ‘soft’ power skills to ultimately transform the decision-making field. The theme on diplomacy when integrated with the street-level bureaucrat theme creates a hybrid framework, which can be a unique lens to explore the role of GPs. The themes highlight the hybrid micropolitical role of GPs in primary care, which this study aims to contribute to the health policy debate about GP in primary care, most especially dementia care.

## Discussion

### Summary

The social theory concept applied to assess power in the findings of this paper is the actor–agency theory of power.^19,20^ The findings of our study align with some of the characterisation of GPs as street-level bureaucrats, and the three themes of discretion, working under pressure, and uncertainty about translating policies and guidelines highlight some street-level bureaucracy characteristics. In addition, the theme of diplomacy describes another skill used by GPs in navigating difficult decisions. This unique combination of the formal (street-level bureaucracy) and informal (street-level diplomacy) strategies used by GPs enhanced their ability to cope with individual encounters and complexities.

### Strengths and limitations

This study was conducted and analysed within an interpretivist paradigm, which recognises that GPs’ accounts will reflect their own perceptions and choices as portrayed to the interviewer. Rather than framing hypothetical clinical scenarios of difficult decisions, GPs explored their experiences in the context of their physical environment (GP surgery, home visit, out of hours, care home) using their own narrative reflections. In addition, choosing purposive sampling allowed the researcher to select a particular mix of participants representing the Yorkshire and Humber region. In so doing, the researcher had some protection from the danger of simply interviewing those who were most willing or most visible. Even though the effects of the pandemic limited recruitment to only the Yorkshire and Humber region, these were patterns of behaviour that appear transferable to other settings.

### Comparison with existing literature

Lipsky’s concept says that street-level bureaucrats are public workers who have a high level of discretion and autonomy in their work: *‘the decisions of street-level bureaucrats, the routines they establish, and the devices they invent to cope with uncertainties and work pressures, effectively become the public policies they carry out’.* ^21^ This concept of street-level bureaucrats has dominated the discussions about how to explain the work GPs do at a micropolitical level and there have been calls from experts in the field for the use of this concept as a way of understanding GPs’ behaviour.^22^ Some argue that the *‘current changes in the wider NHS, with the rise of scientiﬁc-bureaucratic medicine and a public health or population-based concept of health and disease’* has made GPs behave more like street-level bureaucrats as a result of *‘the development of routines and simpliﬁcations’ to help deal with the pressures associated with demand that outstrips supply’*.^13^

However, health systems research studies that solely use the theory of street-level bureaucrats have been criticised for not identifying the intersection between the street-level bureaucrat theory and other theories.^12^ Literature on street-level bureaucracy focuses on the autonomy of frontline actors, ignoring the adaptive skills of diplomacy needed by these actors in complex contexts such as difficult decision making situations.^23^ This study, in addition to identifying GPs as street-level bureaucrats, draws attention to the less talked-about skills of diplomacy that GPs use in carrying out their routine professional responsibilities. This alternative perspective on the conceptualisation of GPs work is captured in a study by Gale *et al* ^10^, where the principles of multi-track diplomacy were adapted to some of the work GPs do. They posit that the soft power of negotiation, persuasion, and relationship building formed the core of how GPs influence decision making,^10^ and other similar arguments support the role of soft power in healthcare.^10,24,25^ The soft power of diplomacy that seeks to transform the decision-making environment is similar to the actor–agency perspective which sees power as an outcome of interactions between social actors.^20^ Understanding this expression of power during policy interaction will enable us appreciate the extent to which GPs shape health decision outcomes.

### Implications for research and practice

Policy makers could better recognise that GPs are acting as creative problem solvers in a situation where guidelines and advanced plans often break down. GPs function as more than just street-level bureaucrats, engaging with various stakeholders in the community to do more than simply implement guidelines. Rather, their skills of diplomacy can be seen as a way of smoothing the implementation of health initiatives and policies.

Before our study, no study in the UK had explored the micropolitics involved in GPs’ decision-making processes in people with dementia. GPs possess and express power and influence over decision-making in people living with dementia when navigating biomedical, social, and psychological factors. This power comes in the form of soft power (street-level diplomacy) and the more formal power of street-level bureaucracy.

## Data

The dataset relied on in this article is available from the corresponding author on reasonable request.
